# Leveraging the ExpandNet framework and operational partnerships to scale-up brief Cognitive Behavioral Therapy in VA primary care clinics

**DOI:** 10.1017/cts.2022.430

**Published:** 2022-07-20

**Authors:** Derrecka M. Boykin, Laura O. Wray, Jennifer S. Funderburk, Steve Holliday, Mark E. Kunik, Michael R. Kauth, Terri L. Fletcher, Joseph Mignogna, Richard B. Roberson, Jeffrey A. Cully

**Affiliations:** 1 HSR&D Center for Innovations in Quality, Effectiveness, and Safety, Michael E. DeBakey VA Medical Center, Houston, TX, USA; 2 VA Center for Integrated Healthcare, Office of Mental Health and Suicide Prevention, Washington, DC, USA; 3 VA Center for Integrated Healthcare, Syracuse VA Medical Center, Syracuse, NY, USA; 4 VISN 17 Primary Care Mental Health Integration, VA Heart of Texas Health Care Network, Arlington, TX, USA; 5 VA South Central Mental Illness Research, Education and Clinical Center, Michael E. DeBakey VA Medical Center, Houston, TX, USA; 6 Rocky Mountain Mental Illness Research, Education, and Clinical Center for Suicide Prevention, Rocky Mountain Regional VA Medical Center, Aurora, CO, USA; 7 Audie L. Murphy VA Hospital, South Texas Veterans Health Care System, San Antonio, TX, USA

**Keywords:** Evidence-based psychotherapy, research–operations partnerships, implementation, cognitive-behavioral therapy, ExpandNet

## Abstract

Evidence-based psychotherapies (EBPs) are underused in health care settings. Aligning implementation of EBPs with the needs of health care leaders (i.e., operational stakeholders) can potentially accelerate their uptake into routine practice. Operational stakeholders (such as hospital leaders, clinical directors, and national program officers) can influence development and oversight of clinical programs as well as policy directives at local, regional, and national levels. Thus, engaging these stakeholders during the implementation and dissemination of EBPs is critical when targeting wider use in health care settings. This article describes how research–operations partnerships were leveraged to increase implementation of an empirically supported psychotherapy – brief Cognitive Behavioral Therapy (brief CBT) – in Veterans Health Administration (VA) primary care settings. The partnered implementation and dissemination efforts were informed by the empirically derived World Health Organization’s ExpandNet framework. A steering committee was formed and included several VA operational stakeholders who helped align the brief CBT program with the implementation needs of VA primary care settings. During the first 18 months of the project, partnerships facilitated rapid implementation of brief CBT at eight VA facilities, including training of 12 providers who saw 120 patients, in addition to expanded program elements to better support sustainability (e.g., train-the-trainer procedures).

## Introduction

Despite their efficacy [1], evidence-based psychotherapies (EBPs) are infrequently used in clinical settings [2–4]. Contributing factors are complex and represent a combination of challenges that can arise at organizational, provider, and patient levels [5–7]. Recent publications argue for the use of implementation-focused methods early in intervention development, with specific recommendations to collaborate with stakeholders to reduce adoption barriers [8,9]. While stakeholder engagement matters during developmental phases, it becomes critical when implementing and disseminating efficacious practices for wider clinical use [9].

Aligning EBPs and their implementation with the needs of health care leaders (i.e., operational stakeholders) can accelerate their uptake into practice [10]. Operational stakeholders are well-positioned to influence health care innovations, given their role in developing and overseeing programs and policy directives, as well as decision-making authority on resource and staffing allocations [11–13]. Because they exist at multiple levels of an organization (e.g., clinic directors, hospital leaders, program officers), researchers have opportunities to collaborate with stakeholders whose responsibilities and goals fit their project aims and outcomes. For example, a project focused on improving delivery of an EBP at one facility might engage local providers, patients, and clinic director as primary stakeholders. Research objectives that target public health initiatives across geographically diverse settings may warrant additional partnerships with health care system leaders, national and regional program managers, and governmental agencies.

Although researchers and operational stakeholders share a broader goal of improving health care practices, their specific objectives and incentives can differ drastically [12]. Operational leaders are charged with facilitating rapid, effective practice changes that address pressing needs within their organizations (such as overseeing staffing, budgeting, and program policies). By contrast, researchers often prioritize generalizable scientific discoveries and use rigorous study designs that can take years to develop, fund, and execute [14]. Despite these practical differences, involving stakeholders in the scientific process can generate directly applicable research [15]. EBP-focused projects that embed activities consistent with health care system processes and target clinically relevant outcomes will have a higher value to stakeholders [10,15].

Emerging literature has identified components of successful research-operations partnerships (e.g., shared agenda, mutual beneficence, ongoing communication [11–13]). However, more information on effective strategies for leveraging these relationships to improve EBP adoption is needed. Frameworks from other disciplines can guide these research–operations collaborations. For example, the World Health Organization’s ExpandNet framework is widely used for developing scalable interventions [9]. Scalability refers to deliberate actions taken to increase dissemination and implementation of empirically tested clinical innovations. ExpandNet is an empirically derived model that has been used with positive effects in health care systems worldwide and includes practical guidance on how researchers and stakeholders can build institutional capacity to sustain a targeted EBP. It includes five scaling-up components: the (1) the innovation (or targeted EBP), (2) resource team, (3) user organizations, (4) external environmental context (such as health care policies, sociopolitical climate, patient needs), and (5) scaling-up strategies. Innovations appropriate for scaling-up will have documented evidence of their feasibility and efficacy. The resource (or project) team is responsible for facilitating wider use of the innovation within user organizations seeking to adopt it. Next, a scaling-up plan is developed that addresses four strategic choice areas – advocacy and dissemination, organizational processes, costs/resources mobilizations, and monitoring and evaluating scaling-up success.

This article presents a case example using ExpandNet to illustrate how research–operations partnerships can be aligned to promote uptake and continued use of EBPs in health care settings. We also provide lessons learned through partnerships with Veterans Health Administration (VA) operational stakeholders.

## Case Example Overview

Leveraging prior clinical trials [16,17] and a unique funding opportunity, our resource team developed a 5-year quality improvement (QI) project in collaboration with VA stakeholders to address a critical delivery barrier related to the infrequent use of EBPs in mental health integrated primary care (PCMHI) clinics. The project was funded through a VA Health Services Research and Development mechanism aimed at enhancing the impact of research-based programs on veteran care. Primary goals were to develop and evaluate procedures to align EBP programs with the intended clinical environments, address delivery challenges associated with practice, and retain flexibility to meet the needs of providers and patients to sustain EBP use.

### The Innovation

Initial efforts focused on implementation of brief Cognitive Behavioral Therapy (brief CBT), an EBP for patients with depression in primary care [16]. A large randomized trial showed that brief CBT, which involves four to six weekly/biweekly individual in-person and/or remotely delivered sessions [18], improved depression symptoms for up to 12 months compared to usual care [16]. Brief CBT was also found to reduce suicidal ideation [19]. A subsequent pragmatic trial documented its implementation potential for wider use [17].

This project aimed to implement brief CBT in two VA Veteran Integrated Service Networks (VISNs) that encompassed eight US states, assess provider adoption, and evaluate its “real-world” effectiveness on depression outcomes. Given the nature of these aims, the project was classified as a QI initiative by the VA Research and Development Office and considered exempt from Institutional Review Board oversight. Below, we describe collaborations with VA stakeholders and processes to pilot an overarching plan for scaling-up brief CBT based on ExpandNet.

### Brief CBT Resource Team

The resource team sought to increase brief CBT use in VA PCMHI clinics. Resources and staff time were mobilized to: (1) maximize support for VA facilities and providers seeking to adopt brief CBT, (2) problem-solve adoption challenges, and (3) advocate for organizational changes to sustain the intervention. Led by the intervention developer (senior author), the team included administrative staff who handled technical assistance and resources, psychologists who served as collaborators and brief CBT consultants, a data programmer/statistician who led program evaluation, and psychology trainees who engaged in various project design and implementation elements. In addition to VA Health Services Research and Development funding, the team’s efforts were externally resourced through the South Central Mental Illness Research, Education, and Clinical Center and VA Office of Academic Affiliations.

### Environmental Context

Timely access to EBPs is a global concern [20]. Mental health disorders, particularly depression, are a leading cause of disability [21]. Unfortunately, most individuals do not receive treatment for various patient (e.g., stigma), provider (e.g., training, organizational support), and systemic reasons (e.g., workforce availability, government reimbursement policies).

### User Organization and Needs

VA is the largest US integrated health care system and a well-recognized leader in mental health innovations. Despite VA’s substantial investment in national EBP training programs, provider adoption remains limited, suggesting the need for additional strategies to support providers with embedding EBPs into their practices [22]. ExpandNet advocates for engaging operational stakeholders to facilitate adoption.

#### Operational stakeholders

During project development, the resource team invited several VA stakeholders to serve on a steering committee to provide feedback on how to align scaling-up with organizational needs. Given project goals and VA infrastructure, a focus on regional stakeholders was prioritized, as these individuals possessed access and direct influence over clinical services and clinical staff for numerous facilities. Stakeholders were approached based on their knowledge of mental health programs, leadership roles, and ability to impact hospital and clinical program participation in brief CBT implementation. Specific partnerships included leaders from VA Center for Integrated Healthcare, which supports VA’s PCMHI programs nationally, as well as mental health directors from two VISNs (16 and 17) who oversaw 15 hospitals and over 100 outpatient clinics. Stakeholders were asked to meet for annual steering committee meetings, with individual meetings held as needed to evaluate emergent opportunities, challenges, and programmatic progress.

### Scaling-Up Plan

**Fig. 1.**
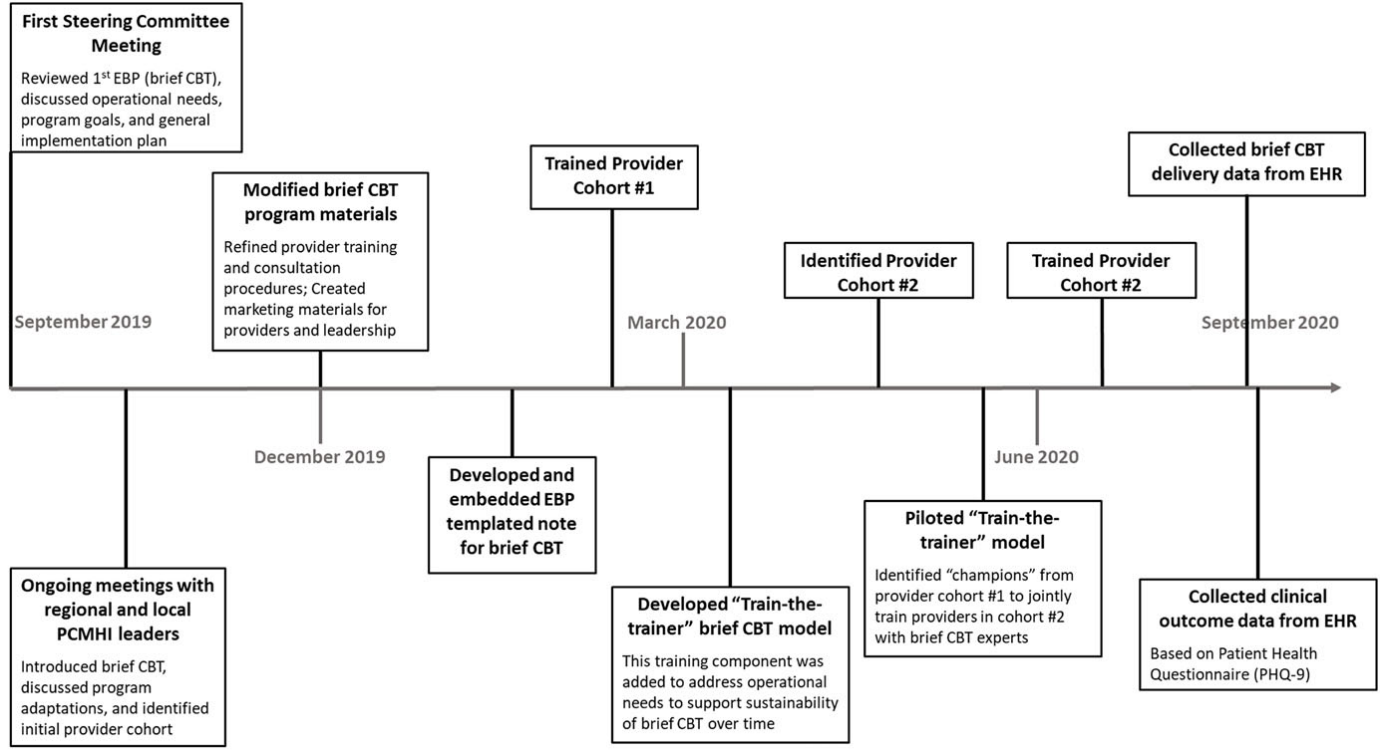
Timeline for partnered implementation of brief CBT in integrated primary care settings in VISN 17 (year 1). *Note*: Brief CBT, Brief Cognitive Behavioral Therapy; EHR, electronic health record.

At the initial steering committee meeting, the resource team and stakeholders discussed plans for scaling-up brief CBT in PCMHI clinics. Clinical data were presented to ensure that stakeholders felt that brief CBT had sufficient evidence to merit its implementation [16,23]. Stakeholders were enthusiastic about its efficacy in addition to its overlap with VA’s initiatives to address depression and suicide [16,23,24]. VISN 17 leaders agreed to implement brief CBT at their eight facilities and to assist the resource team with developing actionable steps to accomplish this. It was decided to delay implementation in VISN 16 to accommodate VISN 17’s needs and to allow the resource team to develop scaling-up procedures to inform future dissemination. While the team planned for a slower rollout over three years, VISN 17 leaders encouraged an implementation start-up within 4–5 months (timeline shown in Fig. 1). Scaling-up plans were formalized through follow-up meetings with VISN leaders, as well as regional and local PCMHI leadership (see Table 1).

**Table 1.** Implementation and scaling-up plan for brief cognitive behavioral therapy (CBT) in integrated primary care mental health (PCMHI) settings based on ExpandNet

Operational needs/priorities	Implementation and scaling-up strategies
*Type of scaling-up: Horizontal approach*	
• Increase use of EBP in PCMHI settings	• Use empirically supported brief CBT intervention and training materials to replicate its implementation in new PCMHI settings. • Add new train-the-trainer processes to brief CBT program.
*Strategic choice 1: Advocacy and dissemination*	
• Promote brief CBT to PCMHI leaders as well as providers at the regional and facility levels to gain “buy-in” for the program.	• Create promotional materials to encourage clinics and providers to participate in the brief CBT program.
*Strategic choice 2: Organizational processes*	
• Request a rapid, flexible implementation of brief CBT across eight US VA facilities in a southern region (VISN 17) • Request sustainable program that could be locally owned.	• Use existing web-based brief CBT provider training program designed to be flexible (tailored training curriculum) and accessible (online). • Provide consultation to providers to address delivery barriers and challenges. • Develop and test train-the-trainer procedures for brief CBT to guide local implementation processes and program sustainability.
*Strategic choice 3: Costs and resource mobilization*	
• Leverage external funding to support brief CBT resource team (e.g., HSR&D, MIRECC, OAA). • Allocate dedicated time (four hours) for providers to participate in brief CBT program. • Use VISN 17 operational resources to create processes to evaluate the brief CBT program (e.g., clinical dashboard).	• Train providers in small cohorts to maximize brief CBT resource team capacity. • Conduct needs assessment with providers and tailor training curriculum to reduce time demands. • Collaborate with VISN 17 regional leaders to develop brief CBT clinical dashboard based on electronic health record data.
*Strategic choice 4: Monitoring and evaluation*	
• Evaluate implementation and clinical outcomes consistent with organizational standards for depression care quality (e.g., adoption rates, number of sessions, patient depression outcomes).	• Embed electronic brief CBT progress note in computerized patient record system to monitor depression care quality and patient outcomes. • Use brief CBT clinical dashboard to monitor delivery and patient outcomes (*under development*).

EBP, evidence-based psychotherapy; PCMHI, Integrated Primary Care Mental Health; Brief CBT, Brief Cognitive Behavioral Therapy; VISN, VA Integrated Service Network (region); HSR&D, Health Services Research and Development Office; MIRECC, Mental Illness Research, Education, and Clinical Center; OAA, VA Office of Academic Affiliations; VA, Veterans Health Administration.

#### Type of scaling-up strategy

A horizontal scaling-up strategy was prioritized, given VISN 17 leadership’s interest in replicating the program at its facilities. The resource team discussed using the existing multifaceted implementation strategy for brief CBT to increase adoption [16]. The implementation strategy included a treatment manual and workbook, a web-based provider training portal, consultation, and tools to facilitate delivery and data collection (e.g., electronic progress note template). Stakeholders supported this implementation approach and asked the resource team and local PCMHI leaders to monitor the program’s success. Other scaling-up strategies were considered but not selected [9]. Vertical scaling-up (e.g., national programing and policy directives) seemed premature, as brief CBT was still emerging as a best practice. Diversification, which involves adding new innovation components, was not pursued as stakeholders preferred to see the intervention replicated as a standalone program.

#### Strategic choice 1. Advocacy and dissemination

The resource team promoted brief CBT to different stakeholders (e.g., PCMHI leaders, clinic directors, providers) to build a broad support base and build institutional mechanisms to facilitate uptake and sustainability. Brochures and handouts were developed and embedded within the program’s website for easy distribution. Stakeholders provided targeted language and strategies to present brief CBT to each stakeholder group. Provider-facing materials described the clinical benefits and professional development opportunities expected from program participation. Materials for PCMHI leaders and clinic directors highlighted intervention alignment with national VA initiatives for depression and suicide prevention. Leadership materials also attended to costs and return on investment.

#### Strategic choice 2. Organizational processes

The resource team adopted a participatory approach to organizational decisions on implementing brief CBT. Modifications to the multifaceted implementation approach were made based on VISN 17’s stated needs and program expectations. As an example, the team created “train-the-trainer” procedures to meet leadership’s request for a decentralized training program that could be “locally” owned. The team agreed to train the first provider cohort and select potential “local” trainers from that cohort who could train subsequent providers. VISN 17 leaders enlisted the assistance of regional PCMHI leaders to assume day-to-day interactions with the team and recruit providers from their hospitals.

#### Strategic choice 3. Costs/resource mobilization

Direct costs related to staff time, information technologies, and programmatic modifications (e.g., train-the-trainer program). To conserve resource team staff time and resources, providers were trained in cohorts using established web-based provider training materials. VISN 17 stakeholders allotted four hours protected time for providers to participate in training and implementation start-up activities. To reduce time burden for providers, brief CBT consultants tailored training based on providers’ CBT knowledge and skills. Providers were also given 2–3 weeks to complete learning modules at their own pace. VISN 17 leaders dedicated technological support toward the creation of a clinical dashboard to evaluate delivery and patient outcomes.

#### Strategic choice 4. Monitoring and evaluation

The resource team identified the processes and outcomes to be monitored to evaluate the “success” of brief CBT implementation. Implementation success was defined as adoption and use of brief CBT by most (≥75%) providers. Stakeholders emphasized the importance of targeting outcomes consistent with VA metrics such as the use of depression symptom measures (i.e., Patient Health Questionnaire-9 [PHQ-9]) and depression care access and quality (e.g., number of brief CBT sessions, number of patients receiving three or more sessions). Aligning program evaluation with VA metrics allowed stakeholders to use these data to advocate for program use by more providers. Automated data collection through an electronic progress note template enabled the resource team to monitor service delivery and clinical impact as they related to these metrics. For audit and feedback purposes [17], the team extracted delivery and clinical data monthly and distributed to program leaders as well as individual providers. Sharing this information with providers during consultation allowed immediate identification of delivery challenges and celebration of successes. In the future, these data will be exported to the clinical dashboard to give mental health leaders and providers real-time information on brief CBT delivery and patient outcomes.

### Preliminary Program Outcomes

Fig. 1 shows the timeline leading up to and following the brief CBT rollout in VISN 17. Input from stakeholders and ongoing interactions with VISN 17 leaders via email, telephone calls, and meetings were essential to enacting the scaling-up plan. During the first 18 months of the project, stakeholders’ total time involvement varied based on duties and project activities. Most members engaged in a minimum of four hours, while others committed 12–16 hours to review documents and attend strategic planning and feedback sessions.

Several months were spent preparing for scaling-up and strengthening relationships with local PCMHI leaders to support dissemination. VISN 17 leaders advocated for providers at each facility to participate, although the final decision was left up to local PCMHI leaders and providers. Train-the-trainer procedures were created to guide local implementation processes and facilitate ownership of the program. Promotional materials were developed and distributed to PCMHI leaders and providers to grow interest in the program.

Beginning January 2020, the resource team trained 15 providers across eight facilities. The first cohort had seven providers, five of whom became local brief CBT “champions” that assisted the resource team with piloting train-the-trainer procedures with a second cohort. These local champions jointly trained eight new providers while learning how to use brief CBT training materials and processes from a trainer’s perspective. Of these 15 providers, 47% were social workers, 40% were psychologists, and 13% were counselors. Twelve (80%) of 15 providers completed training, with nine (75%) continuing to deliver brief CBT. Four providers who left the program transitioned to new positions, while two more had scheduling restrictions that impacted training completion.

Brief interviews with providers assessed their experiences with the program. Providers gave positive remarks about the program’s feasibility and acceptability. They felt intervention and training materials were well developed, user-friendly, and easy to implement. The self-led training approach was “appealing” because training could be completed based on providers’ schedules. Several providers noted how brief CBT’s flexible design for face-to-face and virtual delivery suited needs to deliver care remotely during COVID-19. Some providers reported barriers related to finding time to complete training, feeling “rushed” to cover session content in 30-minute appointments, and tension between brief CBT documentation and clinic-specific reporting standards. As these issues arose, the resource team consulted with operational stakeholders to find solutions, such as encouraging providers to block their schedule for training and streamlining brief CBT documentation to reduce administrative burden.

Preliminary delivery and outcome data suggest that brief CBT was modestly adopted with support for its effectiveness in clinical practice. To date, 12 providers have delivered brief CBT to 120 patients who saw an average of nine (*SD* = 8.13) patients. Traditional intervention fidelity measures such as audiorecorded sessions were not used, but providers documented their administration of core elements of each brief CBT session and use of the PHQ-9 through the electronic progress note template. On the basis of data extracted from these notes, providers appeared adherent to the protocol. Most patients received three or four sessions (*M* = 3.4 ± 2.11), which was associated with a significant reduction in depression (see Table 2). This finding appears superior to previous trials results, although there was no control group to account for regression to the mean. Regardless, these data alleviate concerns of decreases in efficacy of research-developed interventions moved into real-world settings (i.e., voltage drop) [25]. Although only a hypothesis, these clinical outcomes may reflect the autonomy given to providers to engage treatment-seeking patients as opposed to our clinical trials in which patients were randomized without any requirements for treatment seeking. Regular use of PHQ-9 to monitor treatment progress and outcomes can also facilitate symptom improvement [26].

**Table 2.** Preliminary brief CBT for depression patient outcomes relative to prior clinical trial data [16]

	PHQ-9 mean reduction
*Brief CBT clinical trial (N = 180)*	
Patients with depression (PHQ-9 score of 10 or greater)	−3.50 ± 4.86
*HSR&D brief CBT for depression program (N = 120)*	
All patients with depression (PHQ-9 score of 10 or greater)	−4.39 ± 4.95
Patients with depression and three+ brief CBT sessions	−5.22 ± 5.08
Patients with depression and five+ brief CBT sessions	−5.62 ± 5.43

Brief CBT, brief Cognitive Behavioral Therapy; PHQ-9, Nine-item Patient Health Questionnaire; HSR&D, Health Services Research and Development.

A five-point change in PHQ-9 scores indicates a clinically significant reduction.

## Key Lessons Learned

This partnership-based project illustrates an example of a research-operations collaboration that resulted in impactful outcomes for patients across multiple health care systems. The following lessons were identified as important for increasing the public health impact of EBP-focused research, based on our experiences collaborating with stakeholders to rollout brief CBT and achievement of project milestones ahead of the scheduled timeline.

### Lesson 1: Alignment and Engagement

Research–operations partnerships required ongoing communication to ensure alignment with operational priorities through strategic planning using ExpandNet [13]. The initial steering committee meeting established a shared vision and actionable steps to guide scaling-up efforts. The resource team kept monthly and quarterly communication with stakeholders to facilitate discussions about programmatic progress, challenges, and changes to priorities. Maintaining consistent contact was most beneficial for immediately addressing challenges. As the project progressed, a need to transition from formal interactions to informal methods (e.g., emails, telephone calls) was critical to ensure timely communication and prevent delays in implementation. Primary points of contact also shifted during the project, as VISN 17 leaders encouraged the resource team to work with other members of the user organization. Constructing a “business case” (or return on investment proposal) was an effective dissemination strategy to communicate intervention benefits to operational stakeholders. Our business case incorporated stakeholder feedback on topics such as training resources, provider time investment, sustainability of the program locally, and alignment of the program with VA metrics consistent with organizational initiatives (e.g., suicide prevention) and reporting standards (e.g., depression care quality). Promotional materials developed based on this information helped to increase buy-in from clinic leaders and providers.

### Lesson 2: Balancing Competing Demands with Capacity

As the brief CBT program has grown, the resource team has monitored its capacity to support expansions. Scalability remained an important consideration, given the potential to “disappoint” operational stakeholders. The team focused on finding “win-win” solutions while also pursuing “stretch” goals, such as adding train-the-trainer procedures, which were meaningful to stakeholders. Embracing these goals has generated formal opportunities to evaluate the impact of the scaling-up approach as changes are made.

### Lesson 3: Using an Established Framework to Guide Research–Operations Collaborations

ExpandNet enhanced collaboration and shared decision-making by clearly defining the roles of the resource team and operational stakeholders. The team led the implementation process while stakeholders helped to identify actionable steps to achieve the team’s goals and advocated for the program in their clinical systems. Additionally, ExpandNet helped the team focus on elements critical to building an effective implementation and scaling-up plan (e.g., stakeholder engagement, organizational policies). One drawback of ExpandNet is its lack of guidance on implementation outcomes. Moving forward, the team will add components of RE-AIM, which explicitly states how to measure implementation success (e.g., adoption, implementation, maintenance) [27].

### Lesson 4: Embracing Flexible Methodologies for EBP-Focused Programs

Operational needs often demanded flexibility and rapid change to attain outcomes in a time-limited fashion. For example, VISN 17 leaders encouraged rapid implementation of brief CBT across all its facilities. Adopting a flexible project design such as those targeting QI or clinical demonstration enabled the team to make immediate changes to the implementation and scaling-up plans (relative to a randomized controlled trial) [12,13,28]. The team could also respond to new opportunities in real time while not sacrificing demands to maintain rigorous, inflexible scientific methods.

## Conclusions

Implementation and scaling-up approaches that engage operational stakeholders are critical to EBP implementation in health care settings. In this project, ExpandNet helped the resource team maximize the impact of research–operational partnerships. Although targets and methods used to improve care practices will differ across projects, our experiences with stakeholders show the added value of collaboration to rapidly translate research-developed interventions into care. This project moved faster than anticipated, despite challenges (COVID-19); and this success is largely attributable to the stakeholders and alignment of brief CBT to the practical needs of the VA system.

The initial scaling-up procedures provide a firm foundation for implementation of brief CBT at new VA sites. The resource team benefited from having an established multifaceted implementation strategy that could be leveraged in this project. New sustainability elements were added (e.g., train-the-trainer procedures), and their effectiveness will be monitored over time. As brief CBT expands into other regions (e.g., VISN 16), the team will invest in building relationships with VISN leaders and PCMHI stakeholders to tailor implementation to their organizational needs. To strengthen expansion, the resource team and stakeholders are planning for vertical scaling-up by establishing relationships with leaders in national VA program offices (e.g., Office of Mental Health and Suicide Prevention) who can advocate for system-level changes to influence widespread adoption within the larger VA system.

With a shared goal of improving patient care, researchers and operational stakeholders have unique positions to collaborate and address even the most challenging health care improvement initiatives. These collaborations can generate immediate changes and effective, sustainable practices that meet the needs of stakeholders while also accelerating the translational timeline of EBPs. This article highlights several strategies to mitigate tensions that arise from differences in priorities and incentives. As more researchers and stakeholders work together, it will be important to derive clear guidelines for optimizing these partnerships to promote more rapid integration of EBPs into clinical practice.
